# Socioeconomic inequalities in catastrophic health expenditure and impoverishment associated with non-communicable diseases in urban Hanoi, Vietnam

**DOI:** 10.1186/s12939-016-0460-3

**Published:** 2016-10-13

**Authors:** Vu Duy Kien, Hoang Van Minh, Kim Bao Giang, Amy Dao, Le Thanh Tuan, Nawi Ng

**Affiliations:** 1 Center for Population Health Sciences, Hanoi School of Public Health, Hanoi, Vietnam; 2 Institute for Preventive Medicine and Public Health, Hanoi Medical University, Hanoi, Vietnam; 3 Department of Sociomedical Sciences, Mailman School of Public Health, Columbia University, New York, NY USA; 4 Department of Training and Management, Thanh Hoa Medical College, Thanh Hoa, Vietnam; 5 Unit of Epidemiology and Global Health, Department of Public Health and Clinical Medicine, Umeå University, Umeå, Sweden

**Keywords:** Inequality, Catastrophic health expenditure, Impoverishment, Urban, Poverty, Vietnam

## Abstract

**Background:**

The catastrophic health expenditure and impoverishment indices offer guidance for developing appropriate health policies and intervention programs to decrease financial inequity. This study assesses socioeconomic inequalities in catastrophic health expenditure and impoverishment in relation to self-reported non-communicable diseases (NCD) in urban Hanoi, Vietnam.

**Methods:**

A cross-sectional survey was conducted from February to March 2013 in Hanoi, the capital city of Vietnam. We estimated catastrophic health expenditure and impoverishment using information from 492 slum household and 528 non-slum households. We calculated concentration indexes to assess socioeconomic inequalities in catastrophic health expenditure and impoverishment. Factors associated with catastrophic health expenditure and impoverishment were modelled using logistic regression analysis.

**Results:**

The poor households in both slum and non-slum areas were at higher risk of experiencing catastrophic health expenditure, while only the poor households in slum areas were at higher risk of impoverishment because of healthcare spending. Households with at least one member reporting an NCD were significantly more likely to face catastrophic health expenditure (odds ratio [OR] = 2.4; 95 % confidence interval [CI], 1.8–4.0) and impoverishment (OR = 2.3; 95 % CI, 1.1–6.3) compared to households without NCDs. In addition, households in slum areas, with people age 60 years and above, and belonging to the poorest socioeconomic group were significantly associated with increased catastrophic health expenditure, while only households that lived in slum areas, and belonging to the poor or poorest socioeconomic groups were significantly associated with increased impoverishment because of healthcare spending.

**Conclusion:**

Financial interventions to prevent catastrophic health expenditure and impoverishment should target poor households, especially those with family members suffering from NCDs, with older members and those located in slum areas in Hanoi Vietnam. Potential interventions derived from this study include targeting and monitoring of health insurance enrolment, and developing a specialized NCD service package for Vietnam’s social health insurance program.

## Background

Catastrophic health expenditure occurs when out-of-pocket payments for healthcare affect household living expenses. According to World Health Organization (WHO), healthcare expenditures are catastrophic when out-of-pocket payments for healthcare are equal to or exceed 40 % of a household’s capacity to pay or non-subsistence spending, i.e. the income available after basic needs have been met. Impoverishment occurs when a ‘non-poor’ household becomes ‘poor’ after paying for health services [1, 2]. The rates of catastrophic health expenditure and impoverishment due to medical expenses are important indicators for assessing the level of financial protections—in the form of subsidization or health insurance—a country provides for its population. Moreover, these indicators offer guidance for developing appropriate health policies and intervention programs to decrease financial inequity and achieve fairness in financial contribution to the health system [2–5].

Ke et al. estimated that every year, 150 million people experienced catastrophic health expenditures and 100 million people were pushed under the poverty level because of their healthcare payments [4]. This problem was most severe in low- and middle-income countries (LMICs), where most healthcare was mostly paid out-of-pocket [4]. A study in Vietnam showed that the number of households with catastrophic health expenditure and impoverishment increased during the period of 2002–2010 [6]. Kwesiga et al. showed that 25 % Ugandan households experienced catastrophic health expenditure, and about 4 % experienced impoverishment due to health service payments. In Nepal, about 14 % households faced catastrophic health expenditure [7]. The corresponding number among older people households in China was 26 % [8]. Several studies have showed the strong association between having older people as household members with catastrophic health expenditure and impoverishment [2, 4, 9–11].

To date, a multitude of studies have examined catastrophic health expenditure and impoverishment. A search of ‘catastrophic health expenditure’ on PubMed yielded nearly 400 research articles on the topic globally. Most of the studies, however, used country-level population data for measuring catastrophic health expenditure and impoverishment, or focused on the extent of catastrophic health expenditure in relation to specific diseases. Some of the studies compared catastrophic health expenditure and impoverishment between urban and rural areas, and treated the urban population as a single entity [6, 11]. Moreover, the simple delineation of urban versus rural to understand socioeconomic inequality can be misleading, as urban settings can consist of areas with concentrated wealth and concentrated poverty. In many countries with developing economy such as Vietnam, urban areas are expanding rapidly. Rapid urbanization in big cities in Vietnam has led to the presence of the slum areas with poor living conditions that could affect the health of their population [12]. The United Nation defines slum areas as “groups of households where people lived in temporary houses, insecure locations, narrow spaces or nearby/in polluted environment locations” [13]. Generally, health outcomes were worse in slum areas than in urban areas [14–16]. To date, some studies estimated catastrophic health expenditures and impoverishment that focused on the slum areas only [17–20], but no study has assessed if catastrophic health expenditure and impoverishment differ between slum and non-slum areas in the city.

Four main non-communicable diseases (NCD)-- cardiovascular diseases, diabetes, chronic respiratory diseases, and cancer-- account for approximately 63 % of all deaths (or 36 million deaths) worldwide annually [21]. Most of NCD deaths occurred in LMICs [22]. Vietnam currently also suffers an increased burden of NCDs [23]. Due to its chronic nature and the need for long-term treatment, NCDs pose threats to household economics due to increasing health care utilization [24]. Our recent study reported that healthcare utilization was highest among the more well off even within slum populations in urban Vietnam [25]. Many studies indicated that households with members experiencing NCDs faced higher financial risks than households without anyone suffering from NCDs [7, 18, 26, 27]. In this study, we assess socioeconomic inequalities in catastrophic health expenditure and impoverishment associated with self-reported NCDs among household members in slum and non-slum areas in urban Hanoi.

## Methods

### Study design and settings

This study was a part of the research project “The status of health, healthcare utilization and healthcare expenditure of people in urban Hanoi”, which collected information on health-related issues including health behaviors, self-reported NCDs, quality of life, health care utilization, health care expenditure, as well as perception on climate change. In the research project, a population-based cross-sectional survey was conducted from February to March 2013 in Hanoi. Hanoi is the capital city of Vietnam, and the country’s second largest city in term of population and economic development. Hanoi encompasses 30 districts, including 12 urban districts, one district-level town (Son Tay) and 17 rural districts. Each district is divided into wards and towns. As of 2013, Hanoi’s population was estimated to be 6.9 million, of which 2.9 million (42 %) lived in urban districts [28]. In this study, we focused on four urban districts at the center of Hanoi, namely Ba Dinh, Hoan Kiem, Hai Ba Trung and Dong Da districts. These selected urban districts represent typical urban areas with slum and non-slum areas, and they accounted for 1.2 million (41.3 %) of the urban population in Hanoi.

### Sample size

Sample size was calculated using the prevalence of households with any member with self-reported NCDs identified in the pilot survey, which was conducted in a ward of Dong Da district in Hanoi in 60 households. The percentage of households reporting at least one member with NCDs was about 10 %, which was the smallest value as compared to other health indicators. Thus, we selected this figure for our calculation to get enough sample size that covered all other interested health indicators. We used the methods for estimating a population proportion with specified relative precision as proposed by Lwanga and Lemeshow to estimate the sample size [29]. We used the significance level of 0.05 and relative precision of 0.4. After adjusting for an estimated 30 % of non-response and design effects of 2 (in relation to the cluster sampling design), the targeted sample size was at least 600 households in each of the non-slum and slum areas.

### Sampling method

In this study, we defined slum areas as “a group of at least 30 households that are temporary and/or very old houses located in narrow spaces and/or in polluted locations”. Based on this operational definition, we identified a total of 84 slum areas in the four selected districts in Hanoi city. We employed a multi-stage cluster-sampling survey [30]. At the first stage, thirty slum areas were randomly selected from the list of 84 slum areas. For every selected slum area, we selected the adjacent non-slum area in the same ward. At the second stage, twenty households were selected from each of the 60 selected slum and non-slum areas. A household was defined as one person or a group of people who shared accommodation and meals for a period of at least 6 months in the last 12 months. To select households in an area, a household at the center of selected areas was randomly selected as the first household. We moved outward along a street from the center until we identified twenty households in each area. The non-response rate was less than 5 % in both slum and non-slum areas.

**Fig. 1:**
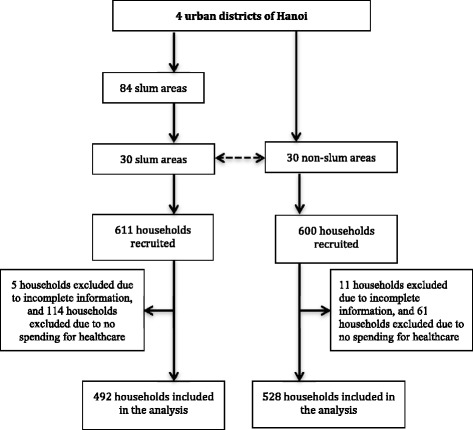
Sampling process in 4 urban district of Hanoi

A total of 1211 households were recruited in this study, of which 1020 households (492 in slum areas and 528 in non-slum areas) with adequate information were analyzed in this study. Figure 1 shows the sampling process in this study.

### Data collections

Face-to-face interviews using a structured questionnaire were conducted with the head of the household. If the head of the household was not present at the time of the survey, we interviewed the spouse or another knowledgeable adult who was available in the household. For this study, we collected information about demographic and socioeconomic of the households, whether any member of households experiences any of the four main NCDs, and household expenditures.

We recruited forty medical students from Hanoi Medical University to conduct the interviews. The interviewers participated in 2 days of training on the study protocol, data collection process, and interviewing skills. We also recruited eight senior staff from the Centre for Health System Research at Hanoi Medical University as supervisors. The supervisors went to the field with the data collectors and checked the completeness of the questionnaires at the end of each day. The supervisors also conducted random rechecks on about 5 % of the households. The interviewers revisited households with incomplete or incorrect information.

### Variables

We used catastrophic health expenditure and impoverishment as two dependent variables in this study. We followed the WHO definitions in developing these two variables [1]. Health expenditure was defined as catastrophic if the household’s out-of-pocket payment for healthcare exceeded 40 % of the household’s capacity to pay. A household was defined as impoverished if a non-poor household became poor, or fall below the poverty line, after paying for healthcare services. In deriving these two dependent variables, we employed the following terms [1]:

- *Household capacity to pay* was defined as a household non-subsistence spending. To estimate household capacity to pay, we subtracted subsistence (or basic necessities) expenditure from the total monthly household expenditure. If households reported food expenditure that was lower than subsistence spending, the non-food expenditure was used as non-subsistence spending.
- *Household subsistence spending* was the minimum requirement to maintain basic standard of living. In this study, we used the household’s average food expenditure in the 45th to 55th percentile adjusted for the household equivalence scale as a proxy measure for subsistence expenditure.
- *Poverty line* was defined by the food expenditure of the household, where the food expenditure share of total household expenditure is at the 50th percentile in the country. Since we did not have a reference from the country’s poverty line, we used the household subsistence (defined above) as a proxy for the poverty line in this study.

The main independent variable for this study was the self-reported NCDs among the household members. The respondents were asked if any of their household members had been diagnosed with NCDs by a doctor or health worker in the last 12 months. In this study, we focused only on four NCDs (including cardiovascular diseases, chronic respiratory diseases, diabetes or cancer) which accounted for 80 % of global mortality [31]. The other independent variables included households living areas (slum or non-slum areas), sex of household head (male or female), household size, presence of older people (>60 years old), presence of children under 6 years, household member participation in social health insurance provided by the government and household socioeconomic status.

### Measurement of socioeconomic status

We used principal component analysis (PCA) to construct the wealth asset index as a proxy for socioeconomic status [32]. Since the slum and non-slum areas had different socioeconomic backgrounds, the PCA scores were estimated separately for the slum and non-slum areas. In the PCA model, we included variables of household’s characteristics such as construction materials (e.g., materials for roofs, walls, and floors), access to utilities and infrastructure (e.g., sanitation facilities and sources of water), and ownership of selected durable assets (e.g., TVs, radios, computers, telephones, refrigerators, washing machines, motorbikes, and cars). We did not include any variables, which reported less than 5 % or more than 95 % of the households. We transformed variables with multiple response categories into a set of dummy variables. Eigenvalues greater than one was used as criteria for extraction, and varimax (orthogonal) rotation was used to improve component interpretation. The PCA resulted in continuous indices separately for the slum and non-slums areas, which were later categorized into wealth quintiles for each area.

### Statistical Methods

To measure the degree of socioeconomic inequality in catastrophic health expenditure and impoverishment, we used concentration index [33]. The formula for concentration index calculation is

1 $$ \mathrm{C}=\frac{2}{\upmu}\mathrm{C}\mathrm{o}\mathrm{v}\left(\mathrm{h},\mathrm{r}\right) $$

where μ is the proportion of catastrophic health expenditure or impoverishment in the study population, h is catastrophic health expenditure or impoverishment of a household, and r is the fractional rank of household in the socioeconomic status distribution [33]. The concentration index ranges between -1 and +1. A concentration index of zero indicates that there is no socioeconomic-related inequality in catastrophic health expenditure or impoverishment in the population. A negative concentration index indicates that catastrophic health expenditure or impoverishment concentrates more among the poor, and a positive concentration index indicates that catastrophic health expenditure or impoverishment concentrates more among the rich.

We carried out all statistical analyses using Stata®13.1. To estimate the concentration index of the catastrophic health expenditure and impoverishment, we used the Distributive Analysis Stata Package (DASP) [34]. The two-tail t-test was used to compare if the values of the concentration index differed from zero.

We conducted descriptive analyses to present the socioeconomic characteristics of the households, out-of-pocket payments for healthcare as a share of household capacity to pay and total household expenditure, as well as the proportion of households with catastrophic health expenditure and impoverishment. We used Chi-square test or Fisher’s exact test to compare the distribution of socioeconomic characteristics, catastrophic health expenditure and impoverishment between households in the slum and non-slum areas. As expenditure was not normally distributed, we used Mann-Whitney test to compare the out-of-pocket payment for healthcare as a share of household capacity to pay and as a share of total household expenditure between households with members experienced NCDs and households without members experienced NCDs. We conducted multiple logistic regression analysis to identify factors associated with catastrophic health expenditure and impoverishment. The level of statistical significance was set to 0.05.

## Results

Table 1 shows the socioeconomic characteristics of study population. The prevalence of households with at least one member with self-reported NCDs in non-slum areas (36.2 %) was significantly higher than that in slum areas (23.6 %). The prevalence of households with at least one member with self-reported cardiovascular disease and diabetes among non-slum households were significantly higher than those observed in the slum areas. The proportions of households with older people 60 years and above, children under 6 years old or all members having health insurance in non-slum areas were significantly higher than those in slum areas.

**Table 1:** Socioeconomic characteristics of households

	Slum, n (%) (*N* = 492)	Non-slum, n (%) (*N* = 528)	*P*-Value (χ^2^ test)
Household with members with self-reported NCDs			
Cardiovascular disease	48 (9.8)	109 (20.6)	<0.001
Chronic pulmonary disease	34 (6.9)	33 (6.3)	0.67
Diabetes	45 (9.2)	79 (15.0)	<0.01
Cancer	11 (2.2)	11 (2.1)	0.87
Any NCD (at least one member with NCDs)	116 (23.6)	191 (36.2)	<0.001
Household with female as household’s heads	239 (48.6)	255 (48.3)	0.93
Household size			
1–2 people	127 (25.8)	76 (14.4)	<0.001
3–4 people	245 (49.8)	231 (43.8)	
≥ 5 people	120 (24.4)	221 (41.9)	
Household with at least one older people ≥60 years old	214 (43.5)	332 (62.9)	<0.001
Household with at least one child <6 years old	129 (26.2)	172 (32.6)	0.03
Household with all members owned social health insurance	223 (45.3)	329 (62.3)	<0.001
Household socioeconomic status (quintile)			
Poorest (20 %)	90 (18.3)	107 (20.3)	0.79
Poor (20 %)	103 (20.9)	121 (22.9)	
Middle (20 %)	99 (20.1)	98 (18.6)	
Rich (20 %)	99 (20.1)	102 (19.3)	
Richest (20 %)	101 (20.5)	100 (18.9)	

*NCDs* non-communicable diseases

### Out-of-pocket payments for healthcare

**Fig. 2:**
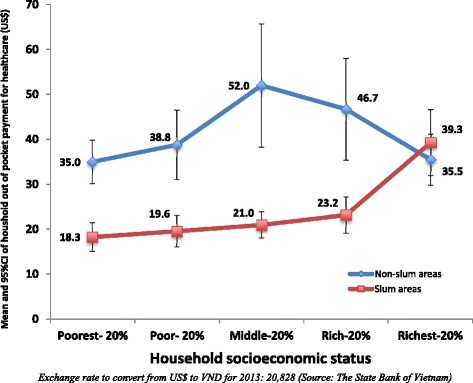
Means and 95 % CI of household out-of-pocket payment per month for healthcare by socioeconomic status

The average monthly out-of-pocket payments for healthcare ranged from US$ 35.0 to 52.0 in non-slum areas and from US$ 18.3 to 39.9 in slum areas. Among the four lowest socioeconomic quintiles, the average monthly out-of-pocket payments for healthcare were significantly lower among slum households than those among non-slum households (Fig. 2). The share of out-of-pocket payments for healthcare to both household’s capacity to pay and total health expenditure were significantly higher among households with at least one member with self-reported NCDs than those among households without any members with self-reported NCDs. When comparing households in slum and non-slum areas, only the share of out-of-pocket payments for healthcare to household’s capacity to pay among households with at least one member with NCDs in slum areas was significantly higher than that among households with at least one member with NCDs in non-slum areas (Table 2).

**Table 2:** Out-of-pocket payment for healthcare as a share of household capacity to pay and total household expenditure

	Overall	Households with at least one member with NCDs	Households without any member with NCDs	*P*-value^a^
Out-of-pocket payment for healthcare as a share of household’s capacity to pay, Median (Interquartile range)				
Slum areas	6.2 (1.8–17.9)	14.8 (5.1–35.2)	4.5 (1.4–15.0)	<0.001
Non-slum areas	6.2 (1.8–14.4)	11.2 (3.2–22.5)	4.5 (1.2–11.0)	<0.001
*P*-value^b^	0.29	0.02	0.21	
Out-of-pocket payment for healthcare as a share of total household expenditure, Median (Interquartile range)				
Slum areas	3.5 (1.0–10.4)	8.3 (3.1–17.3)	2.7 (0.8–8.0)	<0.001
Non-slum areas	3.6 (1.1–9.2)	7.2 (2.0–14.3)	2.9 (0.8–6.7)	<0.001
*P*-value^b^	0.95	0.11	0.63	

*NCDs* non-communicable diseases

^a^Mann-Whitney test to compare between households with at least one member with and without NCDs

^b^Mann-Whitney test to compare between households in slum and non-slum areas

### Pattern and socioeconomic inequalities in catastrophic health expenditure and impoverishment

Generally, the proportion of households facing catastrophic health expenditure in slum areas (10 %) was significantly higher than that in non-slum areas (6.6 %). The pattern was the same with impoverishment in that the proportion of households with impoverishment in slum areas (5.1 %) was significantly higher than that in non-slum areas (1.5 %). In both slum and non-slum areas, the proportion of catastrophic health expenditure among households with at least one member with NCDs were significantly higher than those among households without any member reported NCDs. In slum area, the proportion of impoverishment among households with at least one member with NCDs was significantly higher than that among households without any member reported NCD. Within household with at least one member with NCDs, the proportion of both catastrophic health expenditure and impoverishment among slum households was significantly higher than that among non-slum households. No such differences between slum and non-slum areas were observed among households without any member reporting NCDs (Table 3).

**Table 3:** Pattern of catastrophic health expenditure and impoverishment of households

	Overall	Household with at least one member with NCDs	Household without any member with NCDs	*P*-value^a^
Catastrophic health expenditure, %				
Slum areas	10.0	20.7	6.6	<0.001
Non-slum areas	6.6	10.5	4.5	<0.01
*P*-value^b^	0.05	0.01	0.20	
Impoverishment, %				
Slum areas	5.1	10.3	3.5	<0.01
Non-slum areas	1.5	1.6	1.5	0.94^c^
*P*-value^b^	<0.01	<0.01	0.09	

*NCDs* non-communicable diseases

^a^χ^2^ test to compare between households with at least one member with and without NCDs

^b^χ^2^ test to compare between households in slum and non-slum areas

^c^Fisher’s exact test

Table 4 shows the concentration indices of catastrophic health expenditure and impoverishment. In the overall analysis, all the values of the concentration indices were negative, indicating that the proportion of catastrophic health expenditure and impoverishment concentrated more among the poor households in both slum and non-slum areas. We further stratified the analysis based on whether the households had a least one member with self-reported NCDs. The results showed that the proportion of catastrophic health expenditure significantly concentrated more among the poor households in slum areas in both households with at least member with NCDs (concentration index = -0.30, *p* < 0.001) and households without any member with NCDs (concentration index = -0.37, *p* < 0.01). Among non-slum households, the proportion of catastrophic health expenditure concentrated more among the poor households in only households with at least one member with NCDs (concentration index = -0.31, *p* < 0.01). The proportion of impoverishment significantly concentrated more among the poor households in slum areas in both households with at least member with NCDs (concentration index = -0.42, *p* < 0.001) and households without any member with NCDs (concentration index = -0.36, *p* < 0.001).

**Table 4:** Concentration index of household catastrophic health expenditure and impoverishment

	Overall	Households with at least one member with NCDs	Households without any member with NCDs	*P*-value^a^
Catastrophic health expenditure, Concentration index (SE)				
Slum areas	−0.35 (0.07)***	−0.30 (0.09)***	−0.37 (0.12)**	0.60
Non-slum areas	−0.29 (0.10)**	−0.31 (0.12)**	−0.19 (0.17)	0.54
*P*-value^b^	0.60	0.94	0.38	
Impoverishment, Concentration index (SE)				
Slum areas	−0.40 (0.08)***	−0.42 (0.12)***	−0.36 (0.10)***	0.70
Non-slum areas	−0.23 (0.23)	−0.26 (0.35)	−0.21 (0.30)	0.92
*P*-value^b^	0.50	0.65	0.63	

*NCDs* non-communicable diseases, *SE* standard error

^a^Independent t-test to compare between households with at least one member with and without NCDs

^b^Independent t-test to compare households in slum and non-slum areas within each column

**p < 0.01; ***p < 0.001 (t-test to compare the concentration index with 0)

### Factors associated with catastrophic health expenditure and impoverishment in urban Hanoi

As shown in Table 5, the significant factors related to households experiencing catastrophic health expenditure were households with at least one member reporting diagnosis of NCDs (odds ratio [OR] = 2.4; 95 % confidence interval [CI] = 1.5–3.9), households in slum areas (OR = 2.1; 95 % CI = 1.2–3.5), households with older people (OR = 1.9; 95 % CI = 1.1–3.3), and households belonging to the poorest socioeconomic quintile (OR = 4.9; 95 % CI = 2.0–12.0). The significant factors associated with impoverishment were households with at least one member with self-reported NCDs (OR = 2.3; 95 % CI = 1.1–5.0), households in slum areas (OR = 3.9; 95 % CI = 1.7–9.5), and households belonging to the poor socioeconomic quintile (OR = 11.2; 95 % CI, 1.4–91.4) and the poorest socioeconomic quintile (OR = 9.3; 95 % CI, 1.2–77.8).

**Table 5:** Associated factors of household catastrophic health and impoverishment of households, assessed using multivariable logistic regression analysis

	Catastrophic health expenditure		Impoverishment	
	OR (95 % CI)	*P*- Value	OR (95 % CI)	*P* -Value
Household with at least one member who reported diagnosis of NCDs				
Yes	2.4 (1.5–3.9)	<0.01	2.3 (1.1–5.0)	0.03
No	1		1	
Location of household				
Slum areas	2.1 (1.2–3.5)	<0.01	3.9 (1.7–9.5)	<0.01
Non-slum areas	1		1	
Household with female as household’s heads				
Yes	0.9 (0.6–1.5)	0.78	1.9 (0.9–4.1)	0.80
No	1		1	
Household size				
1–2 people	1		1	
3–4 people	0.7 (0.4–1.3)	0.28	0.6 (0.2–1.4)	0.24
≥ 5 people	0.7 (0.3–1.4)	0.31	0.8 (0.3–2.3)	0.64
Household with at least one older people ≥60 years old				
Yes	1.9 (1.1–3.3)	0.03	0.7 (0.3–1.7)	0.47
No	1		1	
Household with at least one child <6 years old				
Yes	0.6 (0.3–1.2)	0.09	0.9 (0.3–2.5)	0.80
No	1		1	
Household with all members owned social health insurance				
Yes	1.1 (0.6–1.8)	0.82	1.2 (0.6–2.7)	0.67
No	1		1	
Household socioeconomic status (quintile)				
Poorest (20 %)	4.9 (2.0–12.0)	<0.01	11.2 (1.4–91.4)	0.02
Poor (20 %)	2.0 (0.7–5.2)	0.14	9.3 (1.2–77.8)	0.04
Middle (20 %)	2.0 (0.8–5.2)	0.14	6.8 (0.8–59.1)	0.07
Rich (20 %)	1.8 (0.7–4.8)	0.21	3.2 (0.3–33.8)	0.29
Richest (20 %)	1		1	

*NCDs* non-communicable diseases, *OR* Odds Ratio, *95 % CI*: 95 % confidence interval

## Discussion

The findings in this study illustrate the large burden of healthcare expenditure associated with NCDs among households in both slum and non-slum areas in urban Hanoi in Vietnam. We found a higher share of out-of-pocket payments for healthcare among households whose members experienced NCDs. The proportion of catastrophic health expenditure and impoverishment among households whose members experienced NCDs were significantly higher than that among households whose members not experienced NCDs. The poor households (both households with NCDs or without NCDs) in slum areas were significantly more likely to face catastrophic health expenditure and impoverishment. In non-slum areas, only the poor among households whose members experienced NCDs were significantly more likely to face impoverishment because of healthcare spending. We also found that catastrophic health expenditure and impoverishment had significant association between households that had members reporting an NCD, living in slum areas and belonging to the poorest socioeconomic group. These findings provide evidence that NCDs have a significant impact on a household’s financial out of pocket health expenditure for urban settings in Vietnam, and especially for residents in the slum areas.

Disadvantaged household groups, such as households located in slum areas, who are poor, and who have older family member or members suffering from NCDs, are in greater need of health services. At the same time, as the larger proportion of these disadvantaged households pay their health expenses out-of-pocket, they are more likely to be confronted with catastrophic health expenditure and impoverishment [2]. People in slum areas are also disadvantaged as they have lower quality living conditions [35]. Our earlier study in the same setting found that self-reported NCDs were more concentrated among the poor, and being poor is a primary contributor to the increased inequality in self-reported NCDs in the slum areas [36]. With the rapid urbanization and growing size of slum population in Vietnam, it is necessary to address the economic impacts of the slum population, in order to prevent their households being pushed into catastrophic health expenditure and impoverishment [23, 37].

In our current study, we show the differential effects of NCDs on catastrophic health expenditure and impoverishment between the population in slum and non-slum areas. Our findings on the proportion of catastrophic health expenditure and impoverishment in slum Hanoi are comparable with the national-level figures reported in Minh et al.’s study [6]. However, when we take into account whether the households have any members suffering from NCDs, our figures show that the proportion of households with members suffering from NCDs and facing both catastrophic health expenditure and impoverishment are much higher than those reported by Minh et al. Other studies have explored catastrophic health expenditure and impoverishment in urban settings [7, 11, 17, 18, 26, 38], with specific focus on urban areas in general or in slum areas only. Some of these studies show a higher proportion of catastrophic health expenditure and impoverishment among households with NCD patients [7, 18, 26, 27] or households with low socioeconomic status [38].

Although we focused only in the urban areas, our results are consistent with previous studies and show that NCDs and lower socioeconomic status increase the risk of households facing catastrophic health expenditure and impoverishment [8, 39]. Wang et al. found that the concentration indices of catastrophic health expenditure were negative among households with older people with NCDs for both rural and urban areas in China [39]. This means that poorer households with people age 60 years and over with NCDs experienced more catastrophic health expenditure [8].

One unanticipated finding in this study was that health insurance had no association with the reduction of catastrophic health expenditure and impoverishment. Our findings are not in line with some studies conducted in Thailand [18], China [26] and Nigeria [38], which showed that having health insurance decreased catastrophic health expenditure and impoverishment. One potential explanation for the absence of association between health insurance and catastrophic health expenditure and impoverishment in this study might be the increasing consumption of prescription drugs in Vietnam since 2006 [40]. Only 40.8 % of drugs prescribed by doctors followed the essential medicines list. In many cases, patient were asked to pay out-of-pocket for the drugs that were not on the list, which were more expensive and not covered by social health insurance in Vietnam [41]. Another study using the biennial Vietnam Living Standard Survey data between 2002 and 2010 reported that health insurance reduced catastrophic health expenditure only in 2004 and 2006, and impoverishment only in 2004 and 2010 [6]. Several studies also suggested that health insurance in Vietnam had a modest impact on the reduction of catastrophic health expenditure and impoverishment [39, 42–44]. Vietnam has made significant progress towards achieving universal health coverage by expanding the coverage of health insurance for more than 75 % in 2015 [45]. The high proportion of catastrophic health expenditure and impoverishment, however, indicated that there was lack of financial protection for households in urban Hanoi, Vietnam. Expanding the benefits of health insurance is essential in providing financial protection for population in Vietnam [41].

### Strengths and limitations

To our knowledge, this is the first study to examine catastrophic health expenditure and impoverishment for urban households with NCDs in both non-slum and slum areas in Vietnam. We estimated the concentration indices for catastrophic health expenditure and impoverishment to detect their distribution and inequalities among the urban population. These findings may help to identify appropriate population targets for NCDs interventions in an urban setting, particularly in the slum areas in Vietnam. Another strength of this study is the use of the wealth asset index as a proxy for household socioeconomic status. The index was recommended because the asset data used to create the index are more accessible than the income data in many low- and middle-income countries [46].

Our study is limited by its cross-sectional design, which prevents any interpretation about causal relationship. Another limitation was the potential for recall bias, as expenditure data were based on self-reported questionnaire. In addition, the expenditure data was not specific for NCDs, hence the estimates in this study should be interpreted carefully.

## Conclusion

Our study provides evidence that socioeconomic inequalities in catastrophic health expenditure exist in both slum and non-slum areas, while socioeconomic inequalities in impoverishment existed only in slum areas in urban Hanoi, Vietnam. In addition, other conditions such as having a family member with NCDs in the household, living in slum areas, having an older people at home, or belonging to the lowest socioeconomic status increases the odds of a household to face catastrophic health expenditure and impoverishment.

To prevent catastrophic health expenditure and impoverishment, appropriate interventions in terms of improving access to healthcare and removing financial barriers to manage chronic illnesses such as NCDs are essential in urban Hanoi, Vietnam. These interventions should target vulnerable groups, such as households whose members are suffering from NCDs, households in slum areas, households with older people or households in the lowest socioeconomic status. Moreover, the results also suggest that urban policy makers in Hanoi should develop appropriate policies to improve the efficacy of health insurance, such as developing a specialized NCD service package to be included in the health insurance program.
